# Yttrium-90 Radioembolization in Patients with Hepatocellular Carcinoma Who have Previously Received Sorafenib

**DOI:** 10.3389/fonc.2013.00323

**Published:** 2013-12-30

**Authors:** Nitesh Rana, Andrew Wenhua Ju, Michael Bazylewicz, Bhaskar Kallakury, Aiwu Ruth He, Keith R. Unger, Justin S. Lee

**Affiliations:** ^1^Department of Radiation Medicine, Georgetown University Hospital, Washington, DC, USA; ^2^Department of Radiation Oncology, Brody School of Medicine, East Carolina University, Greenville, NC, USA; ^3^Department of Interventional Radiology at Georgetown University Hospital, Washington, DC, USA; ^4^Department of Pathology, Georgetown University Hospital, Washington, DC, USA; ^5^Department of Hematology and Oncology, Georgetown University Hospital, Washington, DC, USA

**Keywords:** yttrium-90, radioembolization, Y90, HCC, hepatocellular carcinoma, sorafenib, SIRT

## Abstract

**Purpose:** Yttrium-90 radioembolization (RE) is a locoregional therapy option for hepatocellular carcinoma (HCC). Sorafenib is a multikinase inhibitor used in HCC that can potentially affect the efficacy of RE by altering tumor vascularity or suppressing post-irradiation angiogenesis. The safety and efficacy of sorafenib followed by RE has not been previously reported.

**Materials and Methods:** Patients with HCC who received RE after sorafenib were included in this retrospective review. Overall survival, toxicity, and maximal radiographic response and necrosis criteria were examined.

**Results:** Ten patients (15 RE administrations) fit the inclusion criteria. All were Barcelona Clinic Liver Cancer (BCLC) stage C. Median follow-up was 16.5 weeks. Median overall survival and radiographic progression-free survival were 30 and 28 weeks, respectively. Significant differences in overall survival were seen based on Child-Pugh class (*p* = 0.002) and radiographic response (*p* = 0.009). Three patients had partial response, six had stable disease, and one had progressive disease. Grade 1 or 2 acute fatigue, anorexia, and abdominal pain were common. Three patients had Grade 3 ascites in the setting of disease progression. Two patients had Grade 3 biochemical toxicity. One patient was sufficiently downstaged following RE and sorafenib to receive a partial hepatectomy.

**Conclusion:** Yttrium-90 RE in patients with HCC who have received sorafenib demonstrate acceptable toxicity and rates of radiographic response. However, the overall survival is lower than that reported in the literature on RE alone or sorafenib alone. This may be due in part to more patients in this study having advanced disease compared to these other study populations. Larger prospective studies are needed to determine whether the combination of RE and sorafenib is superior to either therapy alone.

## Introduction

Hepatocellular carcinoma (HCC) has a global mortality of ~600,000 deaths per year (1). Most patients with HCC present with advanced disease and are poor candidates for curative resection or liver transplantation (2). The options for locoregional therapy to treat unresectable disease include ablative approaches such as percutaneous alcohol injection or radiofrequency ablation, conformal external beam radiation therapy or stereotactic body radiation therapy, and multiple embolization techniques (3). Llovet et al. demonstrated an overall survival benefit for bland arterial embolization or transarterial chemoembolization (TACE) vs. conservative treatment in a randomized trial (4).

Radioembolization (RE) with yttrium-90 (Y-90) microspheres is another locoregional modality for HCC. Y-90 microspheres are engineered to lodge in the microvasculature surrounding liver tumors. The postulated method of action of Y-90 is that liver malignancies draw most of their blood supply from the hepatic arterial system while the normal liver parenchyma draws most of its blood supply from the portal venous system, which allows the microspheres to be preferentially targeted to tumors rather than the normal liver. RE can be delivered with either glass microspheres with an activity of ~2500 Bq per microsphere or resin microspheres with an activity of ~50 Bq per microsphere (5).

Although glass microspheres are FDA-approved for treatment of HCC and resin microspheres are FDA-approved for the treatment of liver metastases from colorectal cancer, both appear to be effective against HCC. A meta-analysis of HCC RE trials found a response rate of 89% for resin Y-90 microspheres, compared to a rate of 78% for glass Y-90 microspheres (6). One large series of HCC patients treated with glass Y-90 microspheres reported an overall survival of 16.4 months (7), while the largest series of patients treated with resin Y-90 microspheres reported an overall survival of 12.8 months (8). RE with glass (9, 10) or resin (11) microspheres appears to have a long-term overall survival that is similar to TACE in unmatched, non-randomized comparisons. One cohort comparison of patients treated with TACE vs. glass microsphere RE reported that RE was superior in downstaging patients with HCC from a United Network for Organ Sharing (UNOS) T3 to UNOS T2, with a rate of 58% compared to 31% (12).

In addition to locoregional therapies, one systemic therapy has recently been approved for the treatment of advanced HCC. Sorafenib is a multikinase inhibitor affecting vascular endothelial growth factor receptor (VEGF), platelet-derived growth factor receptor, and Raf kinase. It has been shown to improve median overall survival by 3 months in patients with advanced HCC in the SHARP trial (13). Sorafenib disrupts tumor vasculature through VEGF and hypoxia-inducible factor-1-alpha (HIF-1-alpha) inhibition (14). Sorafenib may thus potentially decrease the efficacy of RE by reducing the number of microspheres delivered to the tumor. However, it could also theoretically increase the delivery of microspheres to the tumor if radioembolization is performed during the short period of tumor blood flow normalization that follows the initiation of anti-angiogenic therapy (15). In addition, continued use of sorafenib after RE can potentially decrease the induction of intratumor angiogenesis by sublethal irradiation (16). The safety and efficacy of TACE followed in 4–7 days by sorafenib has been demonstrated in the interim analysis of the START trial (17), but the outcomes of RE combined with sorafenib have not been well-reported in the literature. Thirty-four patients who received sorafenib a median of 6 months after RE were included in the series reported by Sangro et al. (8) but their outcomes were not reported separately. The outcomes of patients treated with sorafenib followed by RE have not been previously described. This study examines our institutional experience with HCC patients who received sorafenib followed by Y-90 resin microsphere RE.

## Materials and Methods

IRB approval was obtained for this retrospective review. The records of all of the patients treated at our institution with Y-90 transarterial RE for HCC were reviewed. The patients who had started therapy with sorafenib prior to an administration of RE were identified. Patients were staged according to the Barcelona Clinic Liver Cancer (BCLC) classification system. RE was delivered either in a single unilobar administration or in two separate administrations directed to the right and left lobes for patients requiring bilobar treatment. Patients who started sorafenib between RE administrations for bilobar treatment were included. Patients who were taking sorafenib were asked not to take the drug the day before and the day of RE, and could resume it the day after RE.

All patients were treated with resin Y-90 microspheres. The details for the procedure can be found in the 2006 version of the SIR-Spheres^®^ product insert and in Kennedy et al. (5) A pre-embolization angiogram was performed in all patients, and vessels that supplied the gastrointestinal (GI) tract were coil embolized. A 99m-technecium macroaggregated (99m-Tc MAA) albumin scan was performed to calculate the lung shunt, and a dose-reduction was applied to maintain a lung dose below 30 Gy. The prescribed activity of microspheres to be delivered was calculated using body surface area (BSA) method, which incorporates BSA, liver lobe volume, and percent tumor involvement of the lobe into the dose calculation (5). If stasis was achieved prior to full delivery of the prescribed dose of microspheres, the infusion was stopped and the dose delivered was recorded. A post-embolization Bremsstrahlung SPECT-CT scan was performed to confirm the location of microsphere delivery.

Patients were generally seen in the interventional radiology clinic 1–4 weeks after RE, and were subsequently followed by their medical oncologists. Radiographic imaging (CT or MRI of the abdomen) was obtained serially at the discretion of the treating physician. Radiographic tumor response was evaluated using combined Response Evaluation Criteria In Solid Tumors (RECIST) and necrosis criteria as described by Keppke et al. (18). Only lesions in the treated lobe of the liver were included in the RECIST evaluation. Laboratory values that are examined in detail in this study included serum alpha-fetoprotein (AFP) and serum total bilirubin, although other labs including complete blood count and liver function tests were examined to assess biochemical toxicity. AFP progression was defined as an increase of 20% above the previously measured value, and AFP response was defined as a decrease of 20% below the previous value.

Toxicities were scored using the Common Toxicity Criteria for Adverse Events version 4.0 (CTCAEv4). Constitutional toxicities (fatigue, fever, chills, anorexia, sepsis, and weight loss), GI toxicities (abdominal pain, nausea, vomiting, diarrhea, gastritis, hematochezia, and pancreatitis), liver toxicities (portal hypertension, portal vein thrombosis, and ascites), and lung toxicities (pneumonitis, pulmonary fibrosis, cough, and dyspnea) were included in the assessment of clinical toxicity. If any increase over baseline of the CTCAEv4 grade of a toxicity occurred during the patient’s follow-up, the grade that the toxicity increased to was recorded as the patient’s toxicity. Toxicities were conservatively attributed to RE regardless of whether they could be attributed to be due to RE, tumor progression, or cirrhosis. Acute toxicities were considered to have occurred within 90 days of the last RE. Toxicities that occurred after any subsequent HCC-directed locoregional therapy were censored. As late toxicities occurring >90 days post-RE are less likely to be attributed to RE and more likely due to disease progression or subsequent therapy, they were not recorded as per Sangro et al. (8).

Overall survival and radiographic progression-free survival (PFS) were calculated from time of RE for patients receiving unilobar treatment, and from first RE in the patients who had bilobar treatment. A patient met the endpoint for radiographic PFS if they either died or had progressive disease (PD) on RECIST and necrosis criteria. Survival since time of diagnosis was calculated from time of initial imaging (CT or MRI) or pathology definitive for the diagnosis of HCC. Kaplan–Meier survival between groups was compared using the logrank test. MedCalc^®^ version 11.6.1.0 was used in the statistical analyses. Microsoft Excel 2010 was used for graphing serum AFP and total bilirubin values following RE.

## Results

Ten patients were identified who fit our inclusion criteria. These patients received RE between December 2009 and September 2012. The patient characteristics are described in Table 1. Four patients had disease confined to the liver and six patients had Stage IVA or IVB disease at the time of RE. Seven patients had Child-Pugh class A liver disease, and three patients had Child-Pugh class B liver disease. All 10 patients were BCLC stage C.

**Table 1:** **Patient characteristics**.

**PATIENT CHARACTERISTICS**	
Age (years)	63.5 (56–76)
Total patients (*n*)	10
Gender (*n*)
Male	8
Female	2
Child-pugh class (*n*)
A	7
B	3
BCLC stage (*n*)
C	10
Location of metastases (*n*)
No metastases	4
Lymph node metastases	5
Bone metastases	2
Peritoneal metastases	1
Lung metastases	1
Portal vein thrombosis (*n*)	3
Prior TACE (*n*)	3
Prior hepatectomy (*n*)	1
Sorafenib
Continued use peri-RE (*n*)	9
Sorafenib prior to RE only (*n*)	1
Duration of sorafenib use prior to RE (days)	43 (9–905)

*The median and range or the *n* are shown. A total of 10 patients were treated with RE*.

Nine of the 10 patients were actively taking sorafenib within 2 weeks before at least one RE administration. Two patients initiated their sorafenib therapy between their first and second RE. One patient had stopped sorafenib therapy 5 months prior to the first RE, and then received two cycles of a PARP inhibitor and temozolomide before RE. The doses of sorafenib ranged from 200 mg daily to 400 mg BID. The number of days sorafenib therapy was initiated prior to RE was available for nine patients, with a median of 43 days (range 9–905 days). Three patients had TACE prior to RE, and one patient had received two partial hepatectomies prior to RE. Three patients had portal vein thrombosis prior to RE.

The characteristics of the RE administrations are described in Table 2. RE was performed in a single unilobar administration for five patients. Bilobar treatment in two sessions was performed in the remaining five patients. A total of 15 RE treatments were completed on the 10 patients. The time between the first and second RE for the five patients receiving bilobar treatment ranged from 21 to 97 days. The lung shunts as calculated on the 99m-Tc MAA scan were less than 10% for seven patients. The median total activity delivered per RE was 27.8 mCi (range 10.6–50.6), and the median dose to the target liver lesion(s) was 36.0 Gy (30.3–67.7 Gy). Two of the 15 microsphere administrations had to be stopped due to stasis, with 78 and 91% of the prescribed activity delivered to the liver lobe in these two cases.

**Table 2:** **Treatment characteristics**.

**TREATMENT CHARACTERISTICS**	
Total lobes treated (*n*)	15
Left	7
Right	8
Lobe volume (cc)	1314 (289–2421)
Tumor volume (cc)	231 (16–1576)
Tumor burden (%)	25 (2–70)
Total activity administered (mCi)	27.82 (10.62–50.64)
Dose to liver lobe (Gy)	35.95 (30.33–67.70)
Lung shunt (%)	5 (1–25)
Dose to lung (Gy)	3.46 (0.21–11.73)

*The median and range or the *n* are shown. A total of 15 radioembolizations were completed on 10 patients (*n* = 15)*.

**Figure 1:**
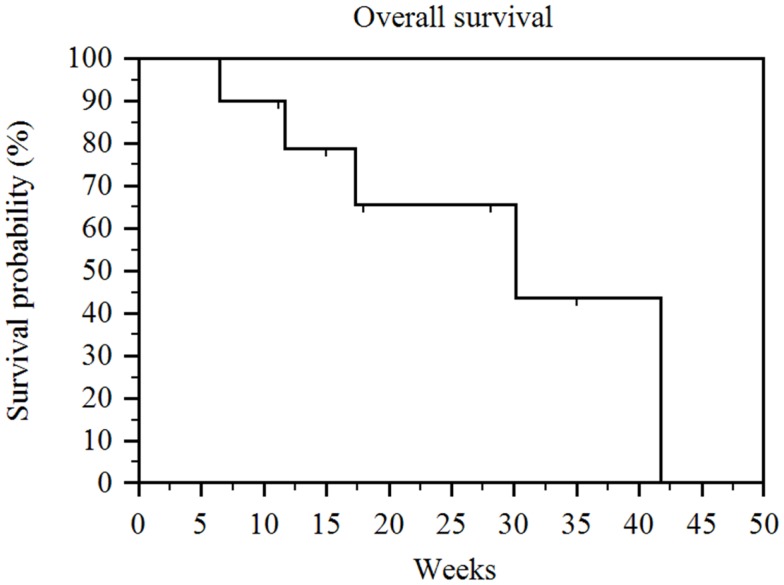
**Kaplan–Meier overall survival**. Median overall survival (measured from time of first RE and including patients censored because they were alive at the time of analysis) for the entire series was 30 weeks (range 6–42 weeks).

**Figure 2:**
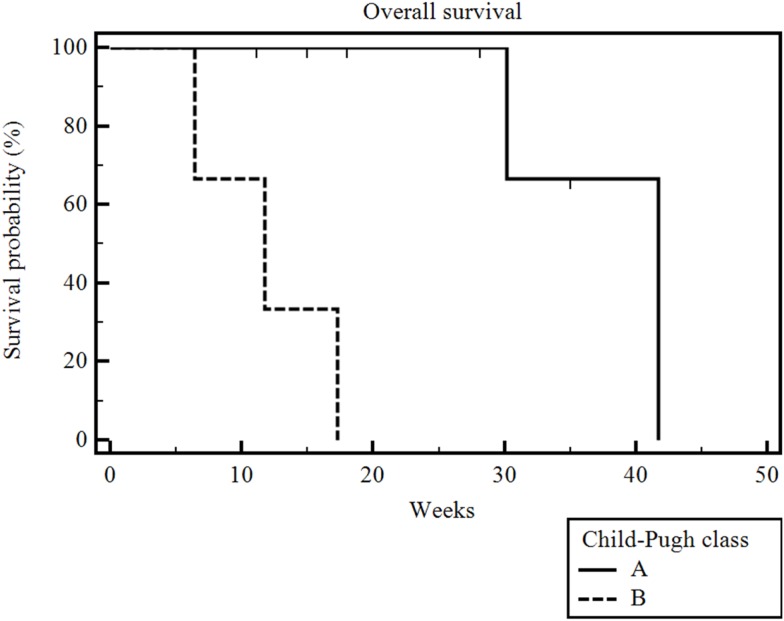
**Kaplan–Meier overall survival by Child-Pugh class**. The median overall survival was 42 weeks (range 11–42 weeks) for Child-Pugh class A patients and 12 weeks (range 6–17 weeks) for Child-Pugh class B patients (*p * = 0.002).

The median follow-up after the first administration of RE was 16.5 weeks (range 6–35 weeks). Five of the 10 patients had died by the time of our analysis. The median overall survival (measured from time of first RE and including patients censored because they were alive at the time of analysis) in our patient population was 30 weeks (range 6–42 weeks) (Figure 1). Of note, the overall survival when measured from time of diagnosis was 65 weeks (range 15–192 weeks) and overall survival when measured from date of initiation of sorafenib therapy was 61 weeks (range 8–183 weeks). The overall survival was significantly correlated with the patient’s Child-Pugh class (*p* = 0.002). The seven patients who had Child-Pugh class A disease had a median overall survival of 42 weeks (range 11–42 weeks), while the three Child-Pugh class B patients had a median overall survival of 12 weeks (range 6–17 weeks) (Figure 2). The median overall survival was not significantly different based on portal vein thrombus vs. no portal vein thrombus (*p* = 0.87), metastatic disease vs. no metastatic disease (*p* = 0.13), prior locoregional therapy vs. no prior locoregional therapy (*p* = 0.47), age >65 vs. <65 (*p* = 0.73), Karnofsky Performance Status >80 vs. ≤80 (*p* = 0.18), bilobar vs. unilobar treatment (*p* = 0.87), or pre-RE AFP >400 vs. <400 ng/mL (*p* = 0.49).

**Figure 3:**
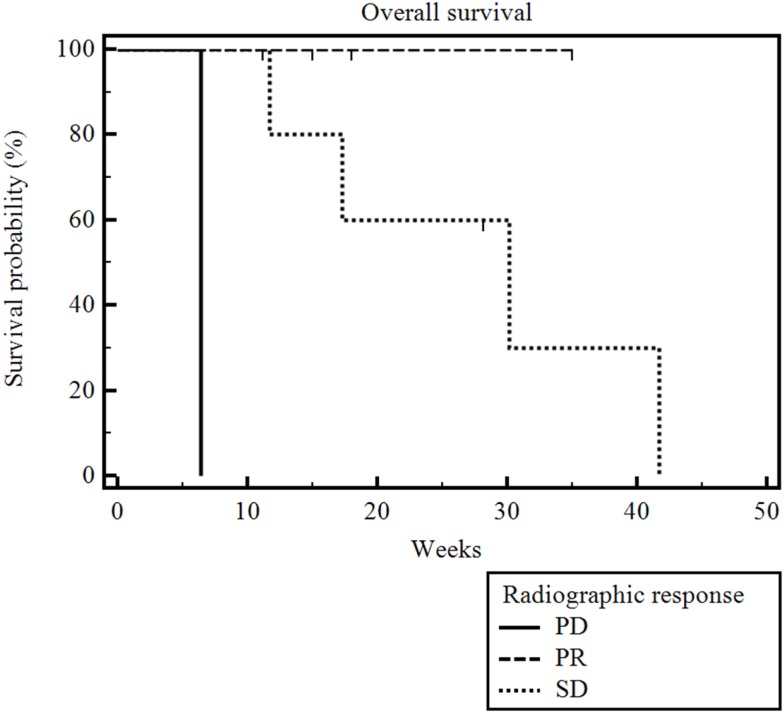
**Kaplan–Meier overall survival by radiographic response**. Median radiographic follow-up was 17 weeks (range 3–35 weeks). The median overall survival was 6 weeks for the one patient with PD, 30 weeks (range 5–28 weeks) for the six with SD, and it was not reached for the three patients with PR (range 6–35 weeks) (*p * = 0.009).

**Figure 4:**
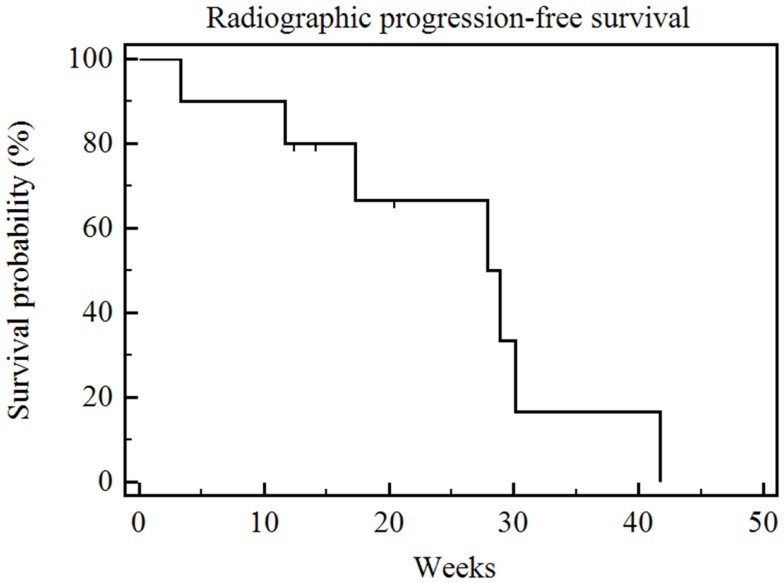
**Kaplan–Meier radiographic progression-free survival**. Median radiographic progression-free survival of the entire series was 28 weeks (range 3–42 weeks).

The median radiographic follow-up was 17 weeks from the time of first RE (range 3–35 weeks). The best radiographic response achieved in the treated liver lobe(s) after RE and prior to the initiation of other systemic or locoregional therapies was partial response (PR) in three patients, stable disease (SD) in six patients, and PD in one patient. The overall survival was significantly correlated with RECIST and necrosis response (*p* = 0.009) (Figure 3). The patient who had PD died 6 weeks after his unilobar RE. The median overall survival was 30 weeks (range 5–28 weeks) for the six patients with SD, and it was not reached for the three patients with PR (range 6–35 weeks). The median radiographic PFS for all 10 patients was 28 weeks (range 3–42 weeks) (Figure 4).

**Figure 5:**
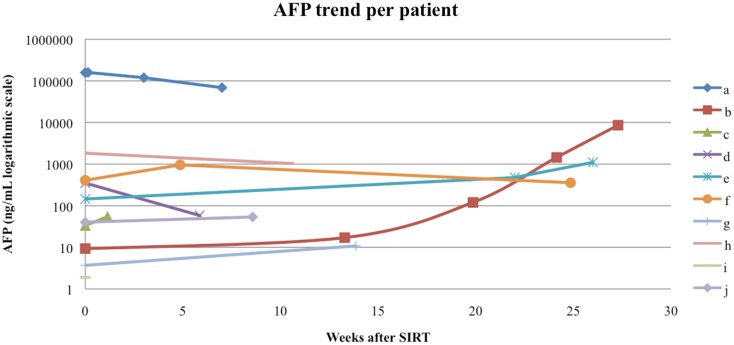
**Alpha-fetoprotein (AFP) trend per patient**. Patients a, f, and h had an elevated pre-RE AFP of >400 ng/mL, all three had a AFP response to RE.

**Figure 6:**
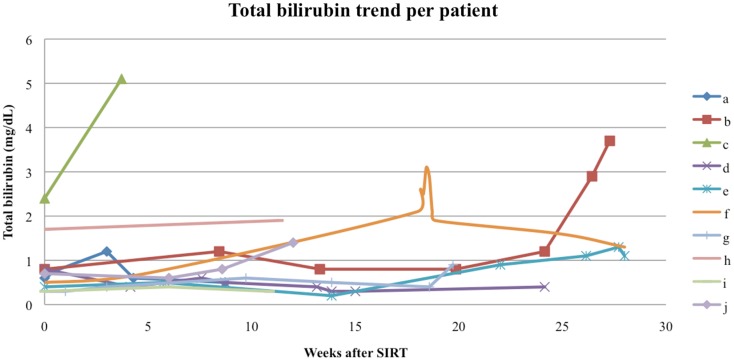
**Total bilirubin trend per patient**. The spike in total bilirubin seen in patient f was secondary to a resection of the remaining tumor mass 18 weeks after RE, patients b and c had total bilirubin rises in the setting of disease progression.

Follow-up serum AFP values were available for 9 out of our 10 patients, with a median interval from the first RE to AFP measurement of 60 days (range 8–154 days). The follow-up AFPs for each patient are shown in Figure 5. Of the three patients who had an elevated serum AFP of above 400 ng/mL before their first RE, all three had a >20% response in AFP levels at the first follow-up measurement. The follow-up serum total bilirubin values are shown in Figure 6. In the acute setting (<90 days post-RE), 9 of 10 patients had stable or downward trends in total bilirubin following RE. The rising trends in total bilirubin in patients b and c occurred in the setting of radiographically documented disease progression. Patient f had a temporary spike in the total bilirubin value 18 weeks after RE at the time of a left hepatectomy.

The acute clinical toxicities experienced by the patients in the study after RE are listed in Table 3. The rate of overall (constitutional, GI, liver, lung) acute toxicity was 70% (Table 3). Three patients experienced Grade 3 clinical toxicity, with one patient experiencing Grade 3 ascites in the setting of clear radiographic progression of his liver disease. Two patients experienced Grade 3 ascites prior to scans showing increased liver tumor size that did not meet the RECIST and necrosis criteria for disease progression, and one of those patients also had Grade 3 anorexia and abdominal pain. Common Grade 1–2 toxicities included fatigue (four patients), anorexia (three patients), weight loss (three patients), and abdominal pain, nausea, vomiting, and diarrhea (each two patients). One patient had Grade 2 portal hypertension, and one patient had Grade 1 cough.

**Table 3:** **Acute clinical toxicities**.

CTCAE	Grade 0	Grade 1	Grade 2	Grade 3	Grade 4	Grade 5
**HIGHEST GRADE OF ACUTE CLINICAL TOXICITY (*n* = 10)**						
Constitutional	4	3	2	1	0	0
Fatigue	6	2	2	0	0	0
Fever	10	0	0	0	0	0
Chills	10	0	0	0	0	0
Anorexia	6	1	2	1	0	0
Sepsis	10	0	0	0	0	0
Weight loss	7	1	2	0	0	0
GI	5	2	2	1	0	0
Abdominal pain	7	2	0	1	0	0
Nausea	8	0	2	0	0	0
Vomiting	8	1	1	0	0	0
Diarrhea	8	2	0	0	0	0
Gastritis	10	0	0	0	0	0
Hematochezia	10	0	0	0	0	0
Pancreatitis	10	0	0	0	0	0
Liver	7	0	0	3	0	0
Portal hypertension	9	0	1	0	0	0
Portal vein thrombosis	10	0	0	0	0	0
Ascites	7	0	0	3	0	0
Lung	9	1	0	0	0	0
Pneumonitis	10	0	0	0	0	0
Pulmonary fibrosis	10	0	0	0	0	0
Cough	9	1	0	0	0	0
Dyspnea	10	0	0	0	0	0
Overall	3	3	1	3	0	0

*The number of patients with their highest CTCAEv.4 Grade toxicity is shown by category. There were no Grade 4 or 5 toxicities*.

The acute biochemical toxicities are shown in Table 4. One patient had a Grade 3 elevation in total bilirubin in the setting of disease progression, and the other patient had a Grade 3 increase in AST following his second RE. In addition, five patients had Grade 1–2 biochemical liver toxicity, and eight patients had Grade 1–2 biochemical hematologic toxicity.

**Table 4:** **Acute biochemical toxicities**.

CTCAE	Grade 0	Grade 1	Grade 2	Grade 3	Grade 4	Grade 5
**HIGHEST GRADE OF ACUTE BIOCHEMICAL TOXICITY (*n* = 10)**						
Liver	3	4	1	2	0	0
ALT	5	4	1	0	0	0
AST	9	0	0	1	0	0
Alk Phos	5	4	1	0	0	0
T. Bili	7	2	0	1	0	0
Hematologic	2	2	6	0	0	0
WBC	7	1	2	0	0	0
Hgb	5	2	3	0	0	0
Plt	5	4	1	0	0	0
Overall	1	2	5	2	0	0

*The number of patients with their highest CTCAEv.4 Grade toxicity is shown by category. There were no Grade 4 or 5 toxicities. ALT, alanine aminotransferase; AST, aspartate aminotransferase; Alk Phos, alkaline phosphatase; T. Bili, total bilirubin; WBC, white blood cell; Hgb, hemoglobin; Plt, platelet*.

**Figure 7:**
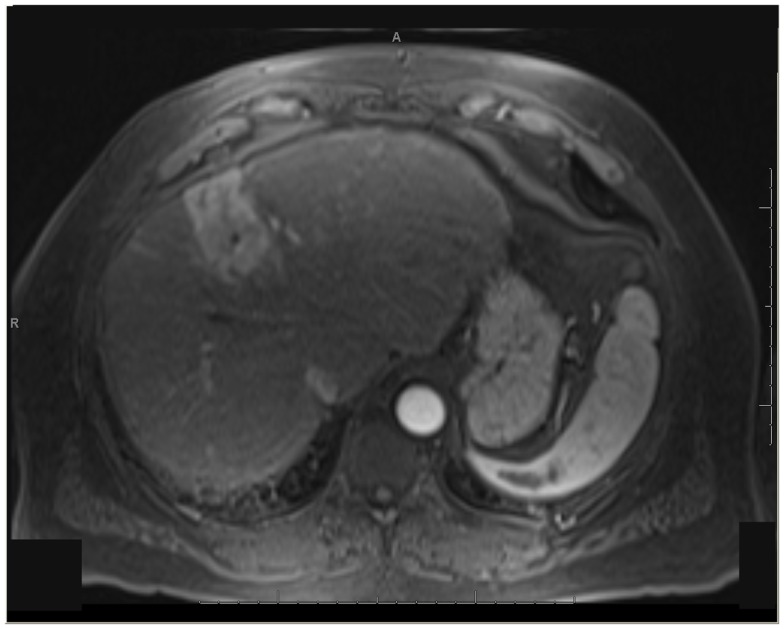
** T1 MRI with early phase contrast**. Pre-radioembolization.

**Figure 8:**
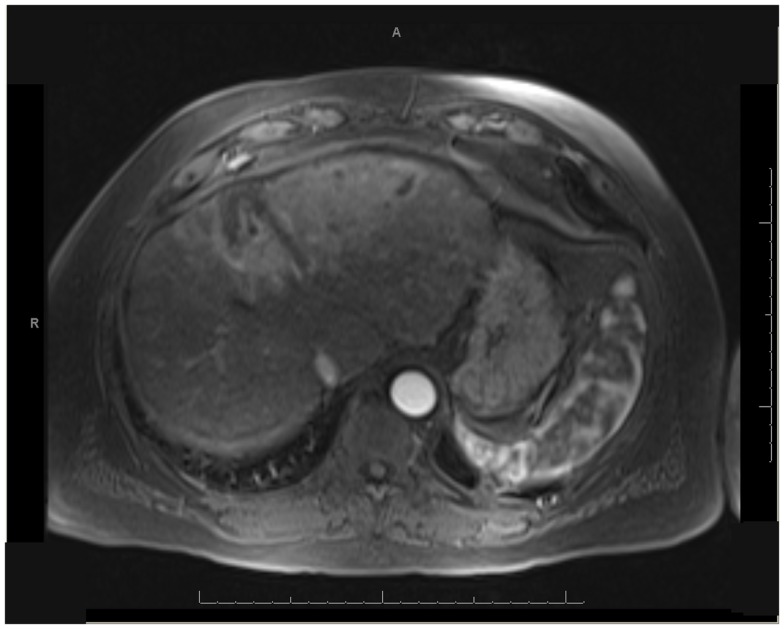
** T1 MRI with early phase contrast**. Seven weeks post-RE.

**Figure 9:**
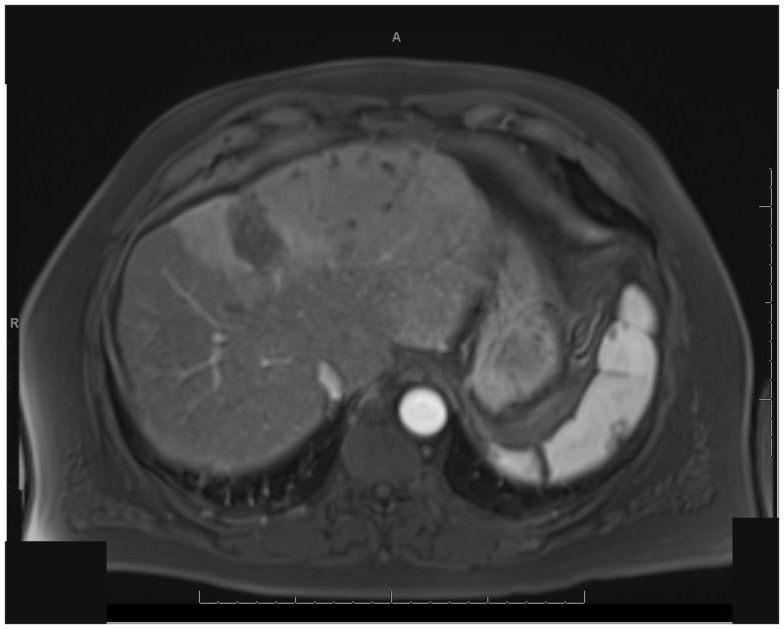
** T1 MRI with early phase contrast**. Fifteen weeks post-RE.

**Figure 10:**
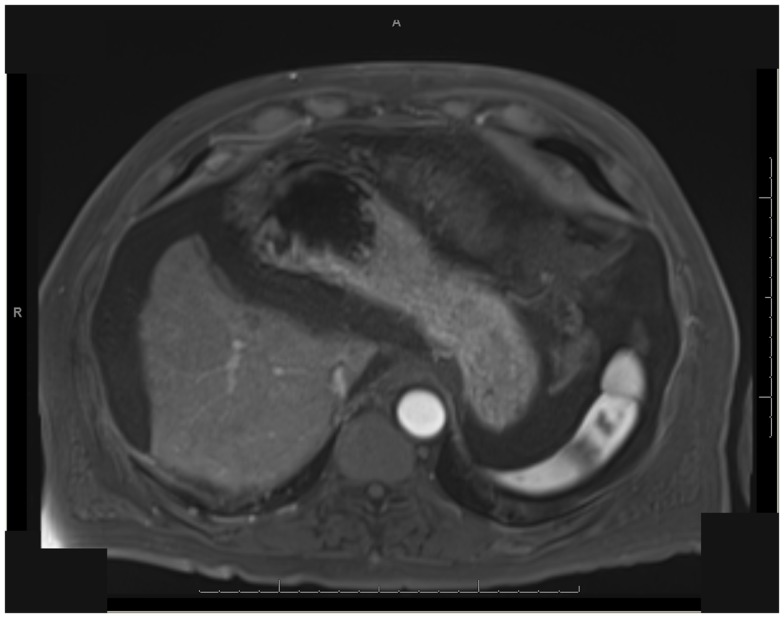
** T1 MRI with early phase contrast**. Status-post extended left hepatectomy, performed 6 months post-RE.

One patient was able to receive a left hepatectomy following RE and sorafenib. This patient was a 60-year-old male with cirrhosis who was diagnosed with HCC in segment IV. He did not meet Milan criteria for liver transplantation due to the 6.7 cm size of the tumor and questionable vascular invasion (Figure 7). He also had non-specific mediastinal lymph node enlargement and a 3 mm right lung nodule at presentation that were suspicious for metastatic disease. He had a left portal vein thrombosis, making him ineligible for TACE. He was Child-Pugh class A, BCLC stage C. He was started on sorafenib at 400 mg BID, and RE with resin Y-90 microspheres was delivered through the left hepatic artery 10 days later. The activity administered was 48.5 mCi, giving a calculated dose of 44.5 Gy to the liver lesion. He experienced mild short-term fatigue, anorexia, and diarrhea following RE. Follow-up imaging from 7 weeks post-RE and 15 weeks post-RE showed significant tumor response (Figures 8 and 9), with well-preserved liver function. He also had an 83% decrease in his serum AFP from 5675 to 957 ng/mL following RE. His sorafenib was stopped 2 weeks prior to the extended left hepatectomy (Figure 10) that the patient received 18 weeks after RE.

**Figure 11:**
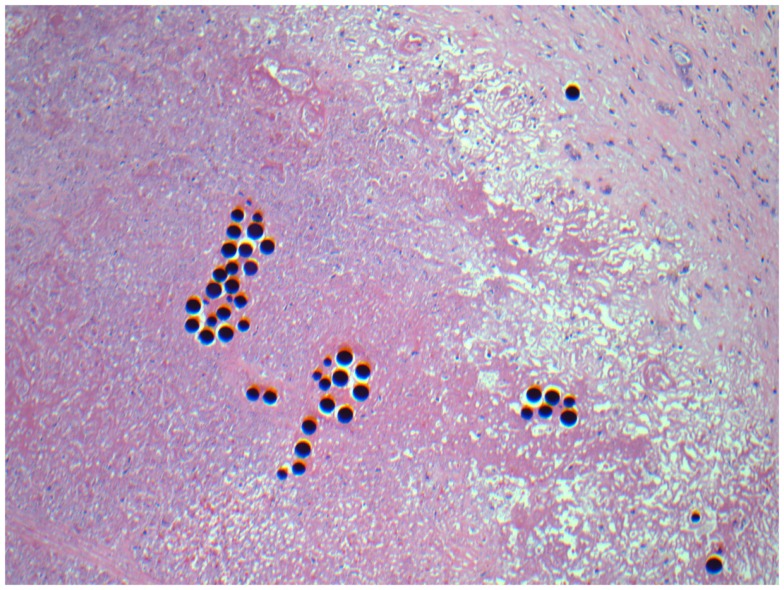
**Micrographs of hematoxylin and eosin-stained sections of the resected liver**. Necrotic tumor with microspheres at 40× magnification.

**Figure 12:**
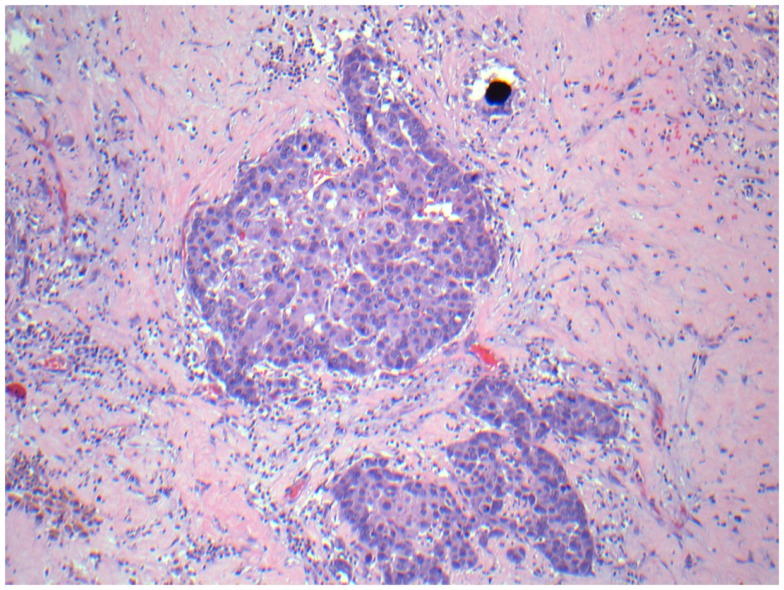
**Micrographs of hematoxylin and eosin-stained sections of the resected liver**. Viable tumor cells with a microsphere at 200× magnification.

**Figure 13:**
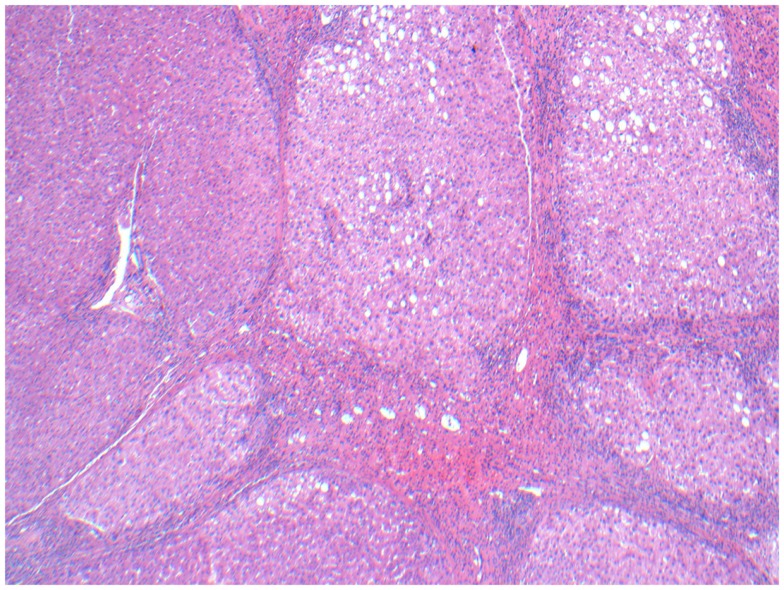
**Micrographs of hematoxylin and eosin-stained sections of the resected liver**. Viable cirrhotic liver tissue with no microspheres at 100× magnification, typical of the patient’s liver that wasn’t involved with tumor.

On surgical pathology, 90% of the resected mass was necrotic (Figure 11). The Y-90 microspheres were more densely localized to the tumor (Figure 12) compared to the uninvolved, cirrhotic liver (Figure 13). Unfortunately, after surgery the patient developed ascites due to liver decompensation, and 2 months after surgery his enlarged subcarinal lymph node was biopsied and showed metastatic HCC. His sorafenib was re-started 4 weeks post-resection. At his last follow-up 8 months after RE, the patient remains on sorafenib and is free of disease in his liver.

## Discussion

In our institutional experience, sorafenib use does not appear to affect the technical feasibility of RE. The procedural aspects of RE, including angiography, coil embolization of the collateral vessels feeding the GI tract, 99m-Tc MAA calculation of the lung shunt, and microsphere delivery were accomplished in these patients without unexpected difficulty, and the prescribed doses were delivered without stasis for the majority of RE administrations. Pathologic examination of one resected specimen suggested that deposition of the microspheres is preferentially directed toward the tumor vasculature rather than the liver parenchyma as expected, but a full dosimetric study of the pathologic specimen could not be performed given the limited amount of tissue that was preserved.

The median overall survival from time of RE was 30 weeks (7 months), compared to a survival since time of diagnosis of 65 weeks (15 months). The overall survival from time of RE for our series is lower than the overall survivals of 12.8 and 16.4 months reported in larger series of patients treated with RE alone (7, 8). This could be due in part to our patients receiving RE late in the course of their disease. In our practice, patients tended to receive other therapies before initiating RE, which is reflected in the difference between the survival from time of diagnosis and the survival from time of RE. In addition, the patients in our study were all BCLC stage C, and our median overall survival from the time of RE is comparable to the 7.3 months reported for BCLC stage C patients in Salem et al. (19). The difference in overall survival between Child-Pugh class A and B patients that was seen in Hilgard et al. (7), Sangro et al. (8), and Salem et al. (19) were also noted in our study. The survival disparity between Child-Pugh class A and Child-Pugh class B suggests that RE could potentially be more effective when performed earlier in the natural course of HCC.

The 12 week overall survival in our three Child-Pugh class B patients is concerning. This low survival is likely due to rapid disease progression, rather than toxicity due to RE and sorafenib. Of these patients, one was on tigatuzumab and sorafenib as part of a clinical trial and stopped tigatuzumab but continued on sorafenib 2 weeks prior to RE due to disease progression. This patient continued to have PD 1 month after completing sequential RE and required additional local therapy with TACE. This patient eventually opted for hospice care and passed away 17.3 weeks after initiation of RE. Another Child-Pugh B patient who had a thrombus in the left portal vein prior to RE had disease progression in the untreated lobe and developed a thrombus in the left hepatic vein 1 month after initiation of RE. This patient elected for hospice care and survived 6.4 weeks following RE. The third Child-Pugh class B patient developed progressive ascites as well as a partial small bowel obstruction of unknown etiology 10 weeks after initiation of RE. This patient elected for hospice care after discharge and passed away 11.7 weeks after RE. Based on the experience of these three Child-Pugh class B patients, RE may not provide benefit in patients with rapid disease progression, and could potentially contribute to their clinical deterioration. Therefore, caution may be warranted in employing RE for BCLC C, Child-Pugh class B patients who are on sorafenib, particularly if their expectation of a response is low.

When compared to treatment with sorafenib alone, the median overall survival from time of RE of 30 weeks (7 months) for our series is lower than the 10.7 months reported in the arm receiving sorafenib in the SHARP trial (13). However, all of the patients included in our series had BCLC stage C disease, vs. 82% of the patients in the experimental arm of the SHARP trial, and 30% of our patients were Child-Pugh class B, vs. 5% in the SHARP trial (13). In addition, overall survival was measured from date of first RE in this study, while it was measured from date of randomization in the SHARP trial. However, the survival in our study from the date of diagnosis is 65 weeks (15 months), and the survival in our study from the date of initiation of sorafenib therapy was 61 weeks (14 months), which compare more favorably to the 10.7 months overall survival reported in the SHARP trial (13).

The radiographic response in our series is comparable to other published results, with 30% having a PR, 60% SD, and 10% PD. Although the meta-analysis by Vente et al. reported an 89 and 78% response rate for resin and glass microspheres respectively, the criteria for response was not well-reported for many of the studies included in the paper (6). The only study in the meta-analysis that reported RECIST response for HCC included only four patients, with 25% PR, 50% SD, and 25% PD (20). Since then, Hilgard et al. have reported that 6% of their cohort had a complete response (CR), 35% PR, 48% SD, and 10% PD by the RECIST and necrosis criteria by 2 months after RE (7). Salem et al. reported a European Association for the Study of the Liver (EASL) response rate of 44% for their BCLC C patients (19). In addition, the radiographic PFS of 28 weeks (6.4 months) for our series is comparable to the 6.0 months time-to-progression reported for BCLC C patients in Salem et al. (19). The 100% AFP response rate in the three patients with pre-treatment AFPs above 400 mg/mL and the ability of one patient to be sufficiently downstaged to have a partial liver resection is promising.

Another possible explanation for lower survival in this study may be due to increase in toxicity. The overall rates of fatigue (54.5–61%), abdominal pain (23–56%), nausea/vomiting (20–32%), anorexia (15%), and fever (3–12.3%) that are reported in the larger series on RE for HCC are comparable to the rates seen in our study (7, 8, 19). However, the rates of Grade 3 clinical toxicity in our study are higher than those reported in these other studies. All of the acute Grade 3 toxicities in our study occurred in the setting of disease progression, which confounds the assessment of whether radiation-induced liver disease contributed to the liver toxicity seen in these patients. All of our patients with Grade 3 acute clinical toxicity experienced Grade 3 ascites, which was not a toxicity examined by Salem et al. or Sangro et al. (8, 19). Hilgard et al. reported a low rate of 3% for Grade 1–2 ascites and no Grade 3 ascites, but the study only included events that occurred within 30 days of RE in their toxicity analysis (7). In general the patients treated in these studies had a lower BCLC stage than the patients in our series, and were thus less likely to develop ascites caused by pre-RE liver dysfunction. The observation that the post-RE levels of serum total bilirubin in our series are stable outside the setting of disease progression or liver resection argues against an increased rate of radiation-induced liver disease. The 40% rate of Grade 2 anemia and thrombocytopenia seen in our study is higher than the 6–15% rate reported in Hilgard et al. and this is most likely related to the continued use of sorafenib after RE (7). There were no Grade 3 or higher hematologic toxicities in our study, compared to the 4% rate of Grade 3 or 4 thrombocytopenia observed in the arm that received sorafenib in the SHARP trial (13).

This study is limited by the small number of patients and the relatively short median follow-up, but the study suggests that combining sorafenib and RE is clinically feasible. Additionally, the ability of one patient in this study to be sufficiently downstaged to have a partial liver resection after sorafenib and RE is promising. However, the shorter survival times in this study, when compared to the survivals reported in the literature on RE alone or sorafenib alone, raises important questions on the safety of combining RE and sorafenib. While the shorter survivals in this study may be explained by patients being further along in their disease course compared to the study populations in the literature, prospective studies are needed to determine whether the combination of RE and sorafenib is superior to either therapy alone. An ideal study would be a randomized controlled trial that compares sorafenib alone, RE alone, and RE+sorafenib. Nevertheless, it may be difficult to accrue enough patients to give such a study sufficient power. Further insight into the number of patients needed to give sufficient power to such a three-arm study may be derived from the ongoing STOP-HCC and SORAMIC trials, which are currently comparing treatment with sorafenib alone vs. RE followed by sorafenib. However, these trials do not employ the concurrent use of sorafenib and RE, and the optimal timing of RE and sorafenib therapy remains an open question.

## Author Contributions

Nitesh Rana: reviewed charts, created database, and authored manuscript. Andrew Wenhua Ju: analyzed data and co-authored manuscript. Michael Bazylewicz: generated combined RECIST and necrosis scores. Bhaskar Kallakury: interpreted pathology slides. Aiwu Ruth He: contributed to discussion on sorafenib. Keith R. Unger: senior author, contributed to discussion on RE. Justin S. Lee: senior author, conceptualized the project and the database, contributed to discussion on radioembolization.

## Conflict of Interest Statement

The authors declare that the research was conducted in the absence of any commercial or financial relationships that could be construed as a potential conflict of interest.
